# Paraneoplastic pemphigus associated with chronic lymphocytic leukemia: a case report

**DOI:** 10.1186/s13256-018-1742-8

**Published:** 2018-08-31

**Authors:** Richard Lucas Konichi-Dias, Aline Fernanda Ramos, Mauricio Eiji de Almeida Santos Yamashita, Cristiane Botelho Miranda Cárcano

**Affiliations:** 1 School of Health Sciences Dr. Paulo Prata (FACISB), Barretos, Sao Paulo Brazil; 2 Barretos Cancer Hospital, Barretos, Sao Paulo Brazil

**Keywords:** Paraneoplastic pemphigus, Chronic lymphocytic leukemia, Respiratory failure

## Abstract

**Background:**

Paraneoplastic pemphigus is a rare multiorgan disease of autoimmune causes, usually triggered by neoplasias, mainly of lymphoproliferative origin, such as leukemia and lymphoma. This disorder is categorized by the presence of autoantibodies that react against proteins, such as desmoplakins, desmogleins, desmocollins, and others that exist in cellular junctions. Paraneoplastic pemphigus can manifest clinically in a variety of ways, ranging from mucositis to lesions involving the skin and pulmonary changes. The diagnosis depends on the correlation between the clinical and histopathologic evaluations. Currently, the treatment of this disease is still very difficult and ineffective. The prognosis is poor, and the mortality rate is very high.

**Case presentation:**

We report a case of a Caucasian patient who had chronic lymphocytic leukemia and developed paraneoplastic pemphigus with severe impairment of skin and mucosa. The initial diagnostic hypothesis was Stevens-Johnson syndrome. The histopathological examination of the skin biopsy was compatible with paraneoplastic pemphigus, and the definitive diagnosis was made on the basis of clinical-pathological correlation.

**Conclusions:**

With the presence of multiorgan lesions in patients with lymphoproliferative neoplasia, paraneoplastic pemphigus should always be considered among the possible diagnostic hypotheses, because diagnosis and early treatment may allow a better prognosis for the patient.

## Background

Paraneoplastic pemphigus (PNP) was first described by Anhalt *et al.* in 1990 in five patients who presented with an atypical form of pemphigus associated with lymphoproliferative diseases [1]. PNP is a very rare disease frequently associated with lymphoproliferative neoplasias (84% of cases), such as chronic lymphocytic leukemia (CLL), non-Hodgkin’s lymphoma, Castleman’s disease, and thymoma [2, 3]. The incidence of the disease is unknown, occurring mostly in adults between ages 45 and 70 years [3]. PNP is triggered by the production of autoantibodies, which form immune complexes that bond to polypeptide chains, mainly desmoglein 1, desmoglein 3, and desmocollin, responsible for cell junction [4].

PNP can manifest clinically in various forms [3]. Severe mucositis accompanied by polymorphic skin lesions frequently appears at the onset of the disease [3]. The lesions tend to be painful, and involvement of mucosal surfaces can occur [3]. The dermatological presentation of PNP can be extremely varied, with dermatologic manifestations that are similar to several diseases such as pemphigus vulgaris, pemphigus bullous, erythema multiform, graft-versus-host disease, and lichen planus [3]. Therefore, the diagnosis of PNP can be confounding [3]. Lung involvement is a prognostic marker reserved for the disease, once it tends to be irreversible during treatment [3].

Many case reports showed that patients with PNP have a poor prognosis, with a mortality rate of 75% to 90%, and the average survival is less than 1 year [5]. The main causes of death are infection and respiratory failure [5].

In PNP, the histopathologic examination is very diverse, including intraepidermal acantholysis (loss of cell-cell adhesion), vacuolar interface dermatitis, and keratinocyte necrosis [6]. Direct immunofluorescence of mucosa and skin biopsies can show deposits of immunoglobulin G and components of the complement throughout the zone of epithelial basal membrane [6]. The diagnosis therefore depends on the correlation between the clinical evaluation and the histopathologic and immunofluorescence examinations.

The first line of treatment is immunosuppression with high doses of systemic corticosteroids [7, 8]. Other immune suppressants used in treatment are cyclophosphamide, mycophenolate mofetil, and azathioprine [8]. There are reports of use of rituximab and alemtuzumab with satisfactory results [7, 8].

## Case presentation

Our patient was a 45-year-old white man. He was a farmer, former alcoholic, and former smoker. He had had high blood pressure for the past 3 years, which was treated with losartan. He had been followed at Barretos Cancer Hospital (BCH) since November 2015 because he had a diagnosis of Binet stage B CLL. Three months after receiving the diagnosis, he developed stage B symptoms and a significant increase of lymph nodes. Because rituximab is not available in our public health system, the patient was treated with fludarabine and cyclophosphamide. He received six cycles of chemotherapy, achieving a partial response. He was followed up, and after 8 months, his disease relapsed, which led to the indication of ibrutinib. However, before the new treatment could be started, he returned to BCH in April 2017, reporting fever and lesions in the oropharynx and skin that had begun 2 weeks prior to this consultation. He reported that he had used penicillin 3 weeks before because of an unrelated condition.

He also said that, in the beginning, the skin lesions were formed by bullae that burst and caused erosions, crusts, and hemorrhagic surface with bloody exudation. He also had oral mucosa and lips lesions. The patient complained of pain in the affected areas and difficulty eating because of the oral lesions. He also reported episodes of a small amount of anal bleeding.

**Fig. 1:**
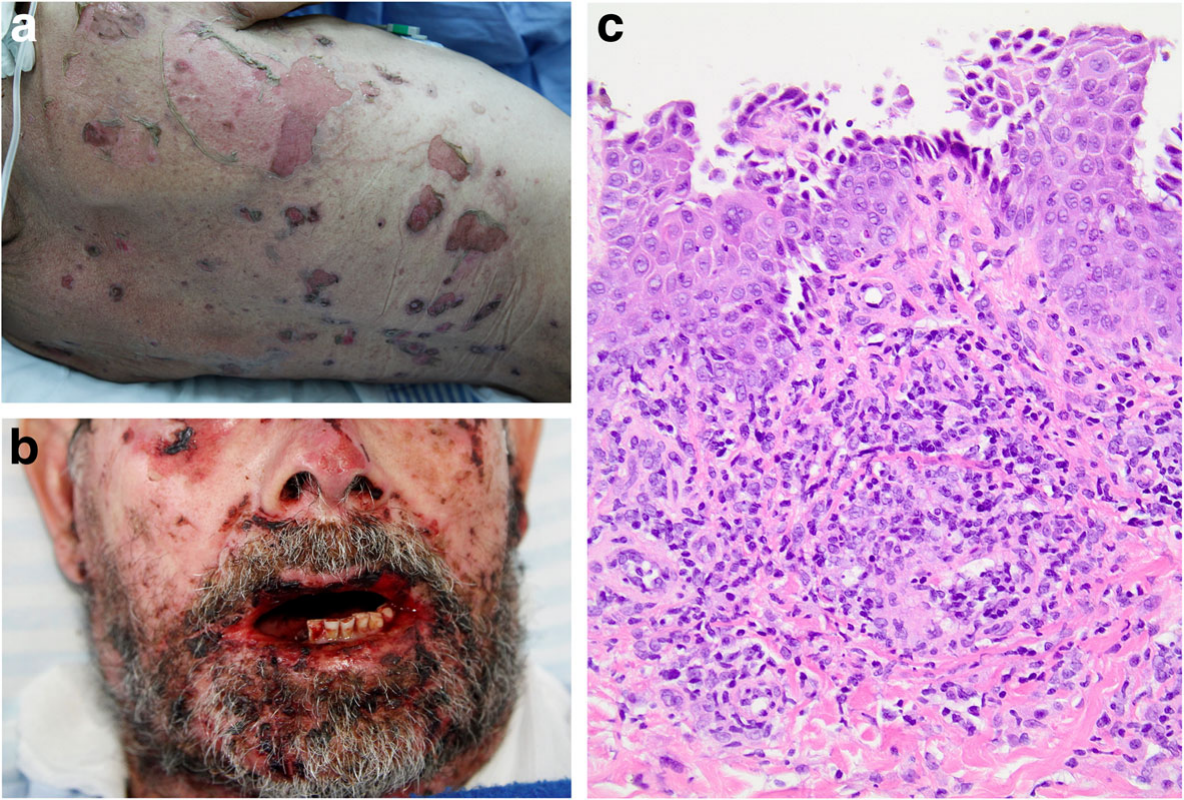
**a** Blisters and extensive erosions of the skin. **b** Severe erosive mucositis of the lips and oral mucosa. **c** Histopathological examination of the biopsy specimen showing keratinocyte apoptosis and acantholysis (hematoxylin and eosin, original magnification × 200)

The patient’s physical examination showed lesions in ocular, oral, and urogenital mucosae; chest; scalp; back; and hand palms. The lesions in the oral mucosa were painful erosions, and hematic crusts were present in the lips. The cutaneous lesions were polymorphic, with bullous erosions, ulcerations, and hemorrhagic crusts involving many areas of the body (Fig. 1a and b). The patient’s Nikolsky sign was positive.

Initially, the main diagnostic hypothesis was Stevens-Johnson syndrome (SJS) and toxic epidermal necrolysis based on the previous report of penicillin use [9]. The patient was directed to the intensive care unit for supportive treatment. Intravenous immunoglobulin was administered for SJS treatment, the lesions were dressed daily, and meropenem and fluconazole were prescribed for a possible secondary skin infection. Although these measures had been taken, the skin and mucosal lesions worsened progressively. The patient’s clinical status deteriorated significantly, which led to the need for oxygen supplementation.

Given the unsatisfactory evolution, biopsies of the skin lesions were performed. The histological analysis showed keratinocyte apoptosis and acantholysis suggestive of PNP (Fig. 1c). Direct immunofluorescence was not performed, owing to unavailability, but based on the clinical-pathological correlation, the diagnosis of PNP was established.

Pulse therapy with methylprednisolone was prescribed for 5 days. There was a discreet initial improvement, but new bubbles appeared in the patient’s face, chest, and limbs, accompanied by intense pain. Four days after the diagnostic confirmation, the patient died of respiratory failure.

## Discussion and conclusions

PNP is a very rare systemic autoimmune disease that was described for the first time by Anhalt *et al.* in 1990 [1]. It is mainly associated with lymphoproliferative neoplasia of B cells, such as CLL and non-Hodgkin’s lymphoma, multiple myeloma, and Castleman’s disease [7]. There have been reports of PNP in sarcoma, lung cancer, and thymoma.

PNP generally causes mucocutaneous alterations with mucositis and polymorphic skin eruptions [4, 10, 11]. The etiology is related to the dysregulation of humoral immune response, and the clinical manifestation involves several organs and occurs by the deposition of immune complexes in the epithelium and mucosa. The involvement of oral mucosa is usually intense, with severe stomatitis and bleeding, and many patients present with erosive and painful conjunctivitis [10]. The skin lesions can be very diverse, and they tend to be disseminated and accompanied by erythema and bullous erosions.

PNP can cause respiratory symptoms such as dyspnea and dry cough due to alterations of respiratory epithelium [11]. In some patients, death by respiratory failure might occur as a result of bronchiolitis obliterans by deposition of immune complexes in the bronchiole epithelium. Death might be caused by various factors, such as sepsis, gastrointestinal bleeding, multiorgan failure, or respiratory failure. The disease is usually progressive and refractory to treatment [11].

PNP can develop in children and adolescents, and unlike the cases reported in adults, it is generally associated with Castleman’s disease (angiofollicular lymphoid hyperplasia) [12]. Castleman’s disease is a rare lymphoproliferative disorder described by important lymph node growth, especially in lymph nodes located in the mediastinum and the retroperitoneum [12].

PNP diagnosis depends on the correlation between clinical characteristics, histological examinations, and direct immunofluorescence [3]. The differential diagnosis includes a broad spectrum of diseases, such as various types of pemphigus, even the drug-induced ones, bullous pemphigoid, bullous drug eruption, lichen planus, graft-versus-host disease, erythema multiforme, SJS, and toxic epidermal necrolysis [3].

In our patient, the disease presented with extensive mucosa and skin commitment from the beginning. However, the patient was first diagnosed with a hypersensitivity reaction to drugs (SJS). This diagnosis was mainly considered because the patient reported the use of penicillin 3 weeks prior to the appearance of the lesions.

Although PNP is a rare disease, any patient who has a neoplasia, particularly a hematological one, that presents with intense stomatitis associated or not with skin lesions must have this diagnosis considered. The aim of treatment of PNP must be the reduction of autoantibody production. Therefore, immunosuppressive therapy must be readily delivered. Nowadays, the first line of treatment is the use of systemic corticosteroids [10]. Corticosteroid-sparing agents can be associated with azathioprine, mycophenolate mofetil, and cyclosporine A. It is fundamental to treat the neoplasias associated with PNP, and the results are better in less aggressive diseases, such as thymoma and Castleman’s disease.

When the first line of treatment fails, other options might be tried. Rituximab, an anti-CD20 monoclonal antibody, can be considered as an alternative in monotherapy or associated with the use of intravenous immunoglobulin [3, 8].

Correct analgesia must be delivered to the patient because the lesions are frequently painful. Patients with PNP have an increased predisposition to skin infections related to the loss of skin integrity. The prognosis is poor, the mortality rates are really high, and death occurs mainly as a result of lung and multiorgan failure or sepsis.

In our patient, histological evaluation was performed through skin biopsy, which showed acantholysis and necrosis of keratinocytes. Direct or indirect immunofluorescence was not performed. However, based on the clinical-pathological correlation, the diagnosis of PNP could be made. The patient died a few days after his diagnosis, even with the use of immunosuppression and broad-spectrum antibiotic therapy for treatment of his skin infection, showing the aggressiveness of the disease, which agrees with data in the literature regarding PNP associated with CLL [5].

PNP is a rare disease that does not have a standard treatment nowadays. This disorder is frequently diagnosed late, because the clinical presentation is similar to that of other more common conditions. However, in lymphoproliferative diseases, PNP might be one of the main diagnostic hypotheses in patients with mucocutaneous damage. Early diagnosis and quick establishment of immunosuppressive treatment are fundamental to the prognosis, but the disease is generally progressive and fatal, especially in cases associated with hematological malignant neoplasia.

## Abbreviations

BCH: Barretos Cancer Hospital
CLL: Chronic lymphocytic leukemia
PNP: Paraneoplastic pemphigus
SJS: Stevens-Johnson syndrome

## References

[1] Anhalt GJ, Kim SC, Stanley JR, Korman NJ, Jabs DA, Kory M, Izumi H, Ratrie H, Mutasim D, Ariss-Abdo L. Paraneoplastic pemphigus: an autoimmune mucocutaneous disease associated with neoplasia. N Engl J Med. 1990;323:1729–35. 10.1056/NEJM199012203232503 2247105

[2] Zimmermann J, Bahmer F, Rose C, Zillikens D, Schmidt E. Clinical and immunopathological spectrum of paraneoplastic pemphigus. J Dtsch Dermatol Ges. 2010;8:598–606. 10.1111/j.1610-0387.2010.07380.x 20180886

[3] Tirado-Sánchez A, Bonifaz A. Paraneoplastic pemphigus: a life-threatening autoimmune blistering disease. Actas Dermosifiliogr. 2017;108:902–10. 10.1016/j.ad.2017.04.024. 28801011

[4] Maverakis E, Goodarzi H, Wehrli LN, Ono Y, Garcia MS. The etiology of paraneoplastic autoimmunity. Clin Rev Allergy Immunol. 2012;42:135–44. 10.1007/s12016-010-8248-5 21246308

[5] Leger S, Picard D, Ingen-Housz-Oro S, et al. Prognostic factors of paraneoplastic pemphigus. Arch Dermatol. 2012;148:1165–72. 10.1001/archdermatol.2012.1830 22801794

[6] Poot AM, Diercks GFH, Kramer D, Schepens I, Klunder G, Hashimoto T, Borradori L, Jonkman MF, Pas HH. Laboratory diagnosis of paraneoplastic pemphigus. Br J Dermatol. 2013;169:1016–24. 10.1111/bjd.12479 23796242

[7] Bech R, Baumgartner-Nielsen J, Peterslund NA, Steiniche T, Deleuran M, d’Amore F. Alemtuzumab is effective against severe chronic lymphocytic leukaemia-associated paraneoplastic pemphigus. Br J Dermatol. 2013;169:469–72. 10.1111/bjd.12324 23517368

[8] Lee A, Sandhu S, Imlay-Gillespie L, Mulligan S, Shumack S. Successful use of Bruton’s kinase inhibitor, ibrutinib, to control paraneoplastic pemphigus in a patient with paraneoplastic autoimmune multiorgan syndrome and chronic lymphocytic leukaemia. Australas J Dermatol. 2017;58:e240–2. 10.1111/ajd.12615. 28295171

[9] Lin YF, Yang CH, Sindy H, et al. Severe cutaneous adverse reactions related to systemic antibiotics. Clin Infect Dis. 2014;58:1377–85. 10.1093/cid/ciu126 24599767

[10] Allen CM, Camisa C. Paraneoplastic pemphigus: a review of the literature. Oral Dis. 2000;6:208–14. 10.1111/j.1601-0825.2000.tb00115.x 10918557

[11] Zhu X, Zhang B. Paraneoplastic pemphigus. J Dermatol. 2007;34:503–11. 10.1111/j.1346-8138.2007.00322.x 17683379

[12] Mimouni D, Anhalt GJ, Lazarova Z, Aho S, Kazerounian S, Kouba DJ, Mascaro JM, Nousari HC. Paraneoplastic pemphigus in children and adolescents. Br J Dermatol. 2002;147:725–32. 10.1046/j.1365-2133.2002.04992.x 12366419

